# Associação entre o Perfil Hemodinâmico da Insuficiência Cardíaca à Admissão Hospitalar e Mortalidade - Programa Boas Práticas Clínicas em Cardiologia

**DOI:** 10.36660/abc.20230699

**Published:** 2024-06-06

**Authors:** André Silva Rodrigues, Fábio Morato de Castilho, Aloisio Joaquim Freitas Ribeiro, Luiz Guilherme Passaglia, Fabio Papa Taniguchi, Antonio L. Ribeiro

**Affiliations:** 1 Universidade Federal de Minas Gerais Serviço de Cardiologia HC Belo Horizonte MG Brasil Universidade Federal de Minas Gerais - Serviço de Cardiologia HC- UFMG, Belo Horizonte, MG – Brasil; 2 Universidade Federal de Minas Gerais Faculdade de Medicina Departamento de Clínica Médica Belo Horizonte MG Brasil Universidade Federal de Minas Gerais - Departamento de Clínica Médica - Faculdade de Medicina – UFMG, Belo Horizonte, MG – Brasil; 3 Universidade Federal de Minas Gerais Departamento de Estatística Belo Horizonte MG Brasil Universidade Federal de Minas Gerais - Departamento de Estatística, Belo Horizonte, MG – Brasil; 4 Hospital do Coração São Paulo SP Brasil Hospital do Coração, São Paulo, SP – Brasil; 5 Universidade Federal de Minas Gerais Centro de Telessaúde Hospital das Clínicas Belo Horizonte MG Brasil Universidade Federal de Minas Gerais - Centro de Telessaúde - Hospital das Clínicas – UFMG, Belo Horizonte, MG – Brasil

**Keywords:** Insuficiência Cardíaca, Mortalidade, Hospitalização

## Abstract

**Fundamento:**

A insuficiência cardíaca (IC) é responsável por alta carga de internações hospitalares. A sua forma de apresentação está relacionada ao prognóstico da doença.

**Objetivos:**

Descrever a associação entre o perfil hemodinâmico de admissão hospitalar na IC aguda, baseado em congestão (úmido ou seco) e perfusão (frio ou quente), e desfechos de mortalidade, tempo de internação e chance de reinternação.

**Métodos:**

Estudo de coorte, envolvendo pacientes do projeto "Boas Práticas Clínicas em Cardiologia", internados por IC aguda em hospitais públicos brasileiros, entre março de 2016 a dezembro de 2019, com seguimento de seis meses. Foram realizadas análises das características populacionais e do perfil hemodinâmico de admissão, além de análises de sobrevivência pelos modelos de Cox para associação entre o perfil de admissão e mortalidade, e regressão logística para chance de reinternação, considerando nível de significância estatística de 5%.

**Resultados:**

Foram avaliados 1978 pacientes, com idade média foi 60,2 (±14,8) anos e fração de ejeção média do ventrículo esquerdo de 39,8% (±17,3%). Houve altas taxas de mortalidade no seguimento de seis meses (22%), com associação entre os perfis hemodinâmicos frios e a mortalidade hospitalar (HR=1,72; IC95% 1,27-2,31; p < 0,001) e em 6 meses (HR= 1,61, IC 95% 1,29-2,02). A taxa de reinternação em 6 meses foi de 22%, sendo maior para os pacientes admitidos em perfis úmidos (OR 2,30; IC95% 1,45-3,65; p < 0,001).

**Conclusões:**

A IC aguda no Brasil apresenta altas taxas de mortalidade e reinternações e os perfis hemodinâmicos de admissão hospitalar são bons marcadores prognósticos dessa evolução.

## Introdução

A insuficiência cardíaca (IC) é uma síndrome complexa, responsável por alta carga de internações hospitalares e mortalidade. É a principal causa de hospitalização entre as doenças cardiovasculares no Brasil, totalizando mais de três milhões nos últimos 10 anos.¹ A taxa de mortalidade hospitalar de pacientes internados por IC aguda no Brasil ainda é muito elevada, atingindo 12,6% no registro BREATHE^2^ de 2015.

Registros internacionais trazem taxas de mortalidade menores, sendo de 5,5% no contexto intra-hospitalar e de 26,7% em um ano após a alta hospitalar, na Europa.^3^ No grande registro americano ADHERE,^4^ a mortalidade hospitalar média no cenário de IC aguda foi de 3,5%. Há diferenças nessas taxas ao se avaliar as características clínicas de perfusão e congestão à admissão hospitalar, com aumento de até 66% na probabilidade de óbito em um ano nos pacientes com perfil frio-úmido em relação aos com perfil quente-seco.^5^

A avaliação clínica de perfusão (pressão arterial, pressão de pulso, perfusão periférica, sensório) e congestão (pressão venosa jugular aumentada, ortopneia, edema, B3) define o perfil hemodinâmico do paciente: quente-seco, quente-úmido, frio-seco e frio-úmido, que se relaciona com a gravidade de evolução do quadro e prognóstico da doença.^6^ Tal classificação foi inicialmente descrita por Diamond-Forrester^7^ em 1976, para pacientes pós-infarto agudo do miocárdio. No contexto de IC, Stevenson LW^8,9^ publicou diversos trabalhos sobre a importância do reconhecimento dos sinais clínicos de congestão e hipoperfusão nos pacientes internados com IC aguda para terapia guiada por perfil hemodinâmico, priorizando a diureticoterapia e vasodilatação naqueles pacientes úmidos e inotrópicos para os pacientes frios.^8,9^

Espera-se que o perfil hemodinâmico de admissão seja um bom marcador prognóstico da IC aguda no Brasil e ajude a caracterizar o atual comportamento dessa doença no país. Ao se associar os perfis de admissão com desfechos de mortalidade e risco de reinternação, intervenções específicas para cada perfil podem ser empregadas para melhoria dos resultados.

## Objetivos

O objetivo do estudo foi demonstrar associação entre o perfil hemodinâmico de admissão hospitalar com desfechos cardiovasculares maiores: mortalidade geral intra-hospitalar (desfecho primário) e mortalidade geral em até 6 meses (hospitalar e seguimento); tempo de internação hospitalar e taxa de reinternação em 6 meses (desfechos secundários).

## Métodos

Trata-se de estudo de coorte, envolvendo pacientes admitidos por IC aguda em 17 hospitais públicos brasileiros, no período de março de 2016 a dezembro de 2019. Os centros são hospitais terciários de todas as regiões brasileiras e participantes do programa Boas Práticas Clínicas em Cardiologia (BPC), da Sociedade Brasileira de Cardiologia (SBC). O BPC é inspirado na iniciativa *Get With The Guidelines* (GWTG) da *American Heart Association* (AHA) e tem como objetivo principal a avaliação das taxas de adesão aos indicadores de desempenho recomendados pelas diretrizes brasileira e americana de cardiologia. O desenho e resultados do programa estão descritos em publicações anteriores.^10-12^

### População

Foram incluídos os pacientes com 18 anos ou mais admitidos com quadro IC aguda (CID-10 I50; I50.0; I50.1 ou I50.9) nos hospitais participantes. Foram excluídos os pacientes com proposta de transferência ou internação prevista menor que 24 horas, além daqueles com outra causa provável para dispneia, mesmo sendo portadores de IC.

### Coleta de dados

Os dados apresentados foram coletados de forma prospectiva durante a internação, usando prontuário eletrônico e formulários próprios, e em 30 dias e seis meses após a alta por meio de contato telefônico feito por entrevistadores treinados. O Termo de Consentimento Livre e Esclarecido foi assinado por todos os participantes e o estudo foi aprovado pelo Comitê de Ética do centro coordenador- HCor São Paulo (sob o número 48561715.5.1001.0060) e no Comitê de Ética do Hospital das Clínicas da UFMG (sob o número 1.487.029).

### Perfis hemodinâmicos

O perfil hemodinâmico foi definido por avaliação clínica do médico responsável pela admissão do paciente, sendo classificado em quente-seco (sem sinais de descompensação); quente-úmido (boa perfusão, mas congesto); frio-seco (má perfusão periférica, mas sem congestão); frio-úmido (má perfusão e congesto). Nessa classificação em quatro categorias, os níveis de exposição definidores são dois: perfusão (frio ou quente) e congestão (úmido ou seco). Baseado nisso, foram feitas análises de sobrevivência e multivariadas para estimar os riscos para os desfechos de interesse para os quatro perfis clássicos, bem como para os padrões de perfusão (perfis frios x perfis quentes) e de congestão (perfis úmidos x perfis secos).

### Análise estatística

Na análise descritiva das variáveis de desfecho e das covariáveis de interesse por perfil hemodinâmico, as variáveis contínuas foram descritas pela média ± desvio padrão ou pela mediana e faixa interquartílica (Q1 e Q3) conforme normalidade dos dados (teste Shapiro Wilk) e as variáveis categóricas foram descritas por suas frequências absoluta e relativa. Os perfis hemodinâmicos foram comparados pelo teste qui-quadrado (variáveis categóricas) e pelo teste de Kruskal- Wallis (variáveis quantitativas). Comparações múltiplas foram realizadas pelo teste de Dunn (variáveis quantitativas) e teste Z para duas proporções (variáveis categóricas), com correção pelo método de Holm, pelo pacote rstatix do software R.

Foram construídas curvas de sobrevivência pelo método de Kaplan Meier, por análise univariada, para avaliar o efeito do perfil hemodinâmico (em quatro e duas categorias) de admissão na mortalidade durante o seguimento do estudo - período de seis meses (teste de log-rank). O grupo de referência para estimativa dos riscos e chances foi o grupo quente-seco.

O modelo de riscos proporcionais de Cox foi utilizado para investigar a influência dos perfis hemodinâmicos sobre a mortalidade durante a internação e sobre a mortalidade no seguimento de seis meses, controlando o efeito das variáveis idade, sexo, presença de comorbidades avaliadas à admissão hospitalar e fração de ejeção do ventrículo esquerdo (FEVE), de forma incremental. Para avaliar a influência dos perfis hemodinâmicos sobre a ocorrência de pelo menos uma reinternação no seguimento de seis meses, utilizou-se modelos de regressão logística, com as mesmas variáveis de controle do modelo de Cox, além do tempo de seguimento dos pacientes após a alta.

O nível de significância adotado na análise estatística foi de 5%. Todas as análises estatísticas foram realizadas nos pacotes *stats, rstatix, survival, survminer, e mice* do *software* R (R Core Team, 2020).

### Falta de dados e tratamento das perdas

O perfil hemodinâmico de 784 (28%) pacientes não foi informado, e eles foram excluídos do estudo. A comparação dos grupos com e sem informação sobre o perfil hemodinâmico quanto ao desfecho primário (morte durante a internação) não revelou diferença significativa entre eles (p-valor = 0,08; Tabela 1 do material suplementar), evidenciando a validade da suposição de perda aleatória dessa informação.

Entre os pacientes com informações válidas sobre o perfil hemodinâmico, 96 possuíam informação faltante para a variável FEVE. Os valores faltantes para essa variável foram imputados pelo método de imputação múltipla, pmm (*predictive mean matching*) disponível no pacote MICE (Multivariate Imputation by Chained Equation) do software R,^13^ resultando em 10 conjuntos de dados completos. Nesse modelo de imputação dos valores de FEVE, foram utilizadas as variáveis sexo, idade, presença de comorbidades, perfil hemodinâmico e uma estimativa do risco de morte durante a internação obtida pelo método de Nelson Aalen. Um total de 679 pacientes não apresentavam dados sobre uso de medicamentos. Como essas variáveis foram utilizadas apenas para a descrição da amostra, seus valores não foram imputados.

Para o desfecho secundário de reinternação em seis meses, foram avaliados 1543 participantes, que são 1.758 sobreviventes menos 215 participantes que não tiveram dois contatos telefônicos efetivos nesse período (12% de perda de seguimento).

## Resultados

### Descrição da amostra à admissão

Dos 2.762 pacientes incluídos na coorte até 2019, foram analisados os dados de 1.978 que tinham a informação do perfil hemodinâmico em prontuário. Os dados basais estão apresentados na Tabela 1, estratificados pelo perfil hemodinâmico de admissão. A população estudada tem idade média de 60 anos, sendo a maioria dos pacientes do gênero masculino, de baixa escolaridade (65% analfabetos ou com ensino fundamental) e baixa renda (73,2% com até dois salários-mínimos de renda familiar mensal). As comorbidades mais relatadas pelos pacientes à admissão foram hipertensão arterial, diabetes mellitus e fibrilação atrial.

**Tabela 1:** Características basais dos pacientes, estratificadas pelo perfil hemodinâmico

Características basais	Total (N=1978)	Quente-seco (n=183)	Quente-úmido (n=1435)	Frio-úmido (n=298)	Frio-seco (n=62)
Gênero masculino* - n (%)	1154 (58,3)	91 (49,7)	832 (58)	191 (64,1)	40 (64,5)
Idade média (DP)	60,2 (14,8)	54,8 (16,7)	61,1 (14,4)	59,2 (14,9)	61,8 (14,4)
**Escolaridade** * **- n (%)**					
Analfabeto	192 (9,7)	16 (8,7)	152 (10,6)	21 (7)	3 (4,8)
Ensino Fundamental	1094 (55,3)	85 (46,4)	817 (56,9)	157 (52,7)	35 (56,5)
Ensino Médio	519 (26,2)	65 (35,5)	353 (24,6)	81 (27,2)	20 (32,3)
Ensino Superior	169 (8,5)	17 (9,3)	112 (7,8)	36 (12,1)	4 (6,5)
**Renda familiar** * **- n (%)**					
<1 salário-mínimo (U$ 270)	656 (33,2)	82 (44,8)	467 (32,5)	93 (31,2)	14 (22,6)
1 a 2 salários (U$ 270- 540)	792 (40)	53 (29)	608 (42,4)	108 (36,2)	23 (37,1)
2 a 5 salários (U$ 540- 1350)	433 (21,9)	39 (21,3)	301 (21)	71 (23,8)	22 (35,5)
> 5 salários (> U$ 540)	90 (4,6)	8 (4,4)	57 (4)	22 (7,4)	3 (4,8)
**Comorbidades autodeclaradas - n (%)**					
Hipertensão arterial*	1351(68,3)	111 (60,7)	1028 (71,6)	178 (59,7)	34 (54,8)
Diabetes mellitus	652 (33)	48 (26,2)	497 (34,6)	92 (30,9)	15 (24,2)
Doença de Chagas*	208 (10,5)	12 (6,6)	135 (9,4)	51 (17,1)	10 (16,1)
Doença coronariana	285 (14,4)	20 (10,9)	217 (15,1)	42 (14,1)	6 (9,7)
Fibrilação/ flutter atrial *	503 (25,4)	30 (16,4)	375 (26,1)	75 (25,2)	23 (37,1)
Doença renal crônica*	309 (15,6)	8 (4,4)	227 (15,8)	60 (20,1)	14 (22,6)
**Classe funcional** * **n (%)**					
I-II	208 (10,5)	67 (36,6)	114 (7,9)	18 (6)	9 (14,5)
III-IV	1393 (70,4)	102 (55,7)	1032 (71,9)	212 (71,1)	47 (75,8)
não informada	377 (19,1)	14 (7,7)	289 (20,1)	68 (22,8)	6 (9,7)
**Fração de ejeção** * **n (%)**					
> 50%	509 (25,7)	77 (42,1)	383 (26,7)	37 (12,4)	12 (19,4)
41-50%	232 (11,7)	24 (13,1)	173 (12,1)	23 (7,7)	12 (19,4)
≤ 40%	1135 (57,4)	66 (36,1)	808 (56,3)	224 (59,7)	37 (59,7)
Insuficiência cardíaca prévia - n (%)	1578 (79,8)	147 (80,3)	1131 (78,9)	243 (81,5)	57 (79,8)
Internações últimos 6 meses*- n (%)	651 (32,9)	65 (35,5)	444 (30,9)	124 (41,7)	18 (29,1)
**Medicamentos de uso Prévio - n (%)**†					
Betabloqueador*	926 (71,3)	75 (61)	676 (71)	147 (77)	28 (84,8)
IECA ou BRA	854 (65,7)	80 (65)	616 (64,7)	137 (71,7)	21 (63,6)
Espironolactona*	524 (40,3)	45 (36,6)	348 (36,6)	109 (57,1)	22 (66,7)
Diurético de alça*	885 (68,1)	72 (58,5)	648 (68,1)	137 (71,7)	28 (84,8)

* p valor < 0,05 para diferença entre os perfis hemodinâmicos (teste qui-quadrado).

† N= 1299 por perda de dados sobre o uso de medicações prévias;

IECA: inibidor da enzima conversora de angiotensina; BRA: bloqueador do receptor de angiotensina.

A maioria dos pacientes já apresentava diagnóstico prévio de IC (IC descompensada) e estava em classe funcional III-IV (*New York Heart Association*, NYHA). As etiologias mais frequentes encontradas na internação (após a realizações de exames e definição pela equipe assistente) foram idiopática (23,3%); isquêmica (21,3%); hipertensiva (16,1%); valvar (15,3%) e cardiomiopatia chagásica (9,9%), dentre outras (14,1%). (Tabela 2 do material suplementar). A maioria dos pacientes do estudo apresentava IC com FEVE (≤ 40%), com valor médio de 39,8% (±17,3%) e o perfil hemodinâmico mais frequente na admissão foi o quente-úmido.

### Análise univariada dos desfechos de interesse

Houve 220 óbitos intra-hospitalares, o que corresponde à uma mortalidade geral de 11,1% da amostra. Essa mortalidade foi maior nos pacientes admitidos com baixa perfusão periférica (perfis frios), sendo de 21,5% no perfil frio-úmido e 14,5% no perfil frio-seco. Apenas 10 pacientes (0,5% da amostra) realizaram transplante cardíaco na internação. Durante o período do estudo, que incluiu a internação e o seguimento em seis meses, a taxa de mortalidade geral foi de 22%, sendo maior nos pacientes admitidos em perfil frio-úmido, que apresentaram taxa de mortalidade geral de 32%. O tempo de internação também variou significativamente entre os perfis hemodinâmicos, sendo a mediana de 17 (9-33) dias na população geral e de 23 (13-42) dias no perfil frio- úmido (Tabela 2).

**Tabela 2:** Desfechos primários e secundários, estratificados pelo perfil hemodinâmico

Desfechos	Total (N=1978)	Quente-seco Grupo 1 (n=183)	Quente-úmido Grupo 2 (n=1435)	Frio-úmido Grupo 3 (n=298)	Frio-seco Grupo 4 (n=62)	p-valor
Óbitos hospitalares N (%)	220 (11,1)	14 (7,7)	133 (9,3)	64 (21,5)	9 (14,5)	<0,001 (1=2≠3=4)
Óbitos período total (internação e 6 meses de seguimento)- N(%)	432 (22)	32 (16)	290 (20)	96 (32)	14 (22)	<0,001 (1=2≠3=4)
Dias de internação Mediana (intervalo interquartil)	17 (9-33)	22 (10-35)	16 (8-30)	23 (13-42)	15 (10-32)	<0,001 (1≠2=3=4)
Reinternação*- N(%)	334 (22)	18 (11,5)	262 (23)	48 (23,8)	6 (13)	0,004 (1≠2=3≠4)

* N=1543 por perdas de seguimento (óbitos na internação e pacientes não acompanhados no seguimento).

No seguimento de seis meses após a alta hospitalar, foram acompanhados 1543 pacientes com dois contatos telefônicos. Desses, 334 (22%) pacientes tiveram pelo menos uma reinternação hospitalar e houve efeito significativo do perfil hemodinâmico de admissão hospitalar para esse desfecho. Nesse caso, a reinternação foi mais frequente nos pacientes congestos (perfis úmidos), sendo 23% no grupo quente-úmido e 23,8% no grupo frio-úmido, e somente 11,5% no grupo quente-seco e 10,9% no grupo frio-seco (Tabela 2).

Na análise de sobrevida univariada pelas curvas de Kaplan Meier, houve associação entre o perfil clínico de admissão e a mortalidade no período total (hospitalar e seguimento em 6 meses), com maior mortalidade do perfil frio-úmido em relação aos outros (p < 0,0001, teste de log-rank). Ao se avaliar separadamente perfusão e congestão, nota-se que os perfis frios tiveram maior mortalidade no seguimento em comparação aos perfis quentes (p < 0,0001, teste de log-rank), o que não foi observado para os perfis úmidos versus secos, p = 0,08 (teste de log-rank). (Figura Central).

**Figure:**
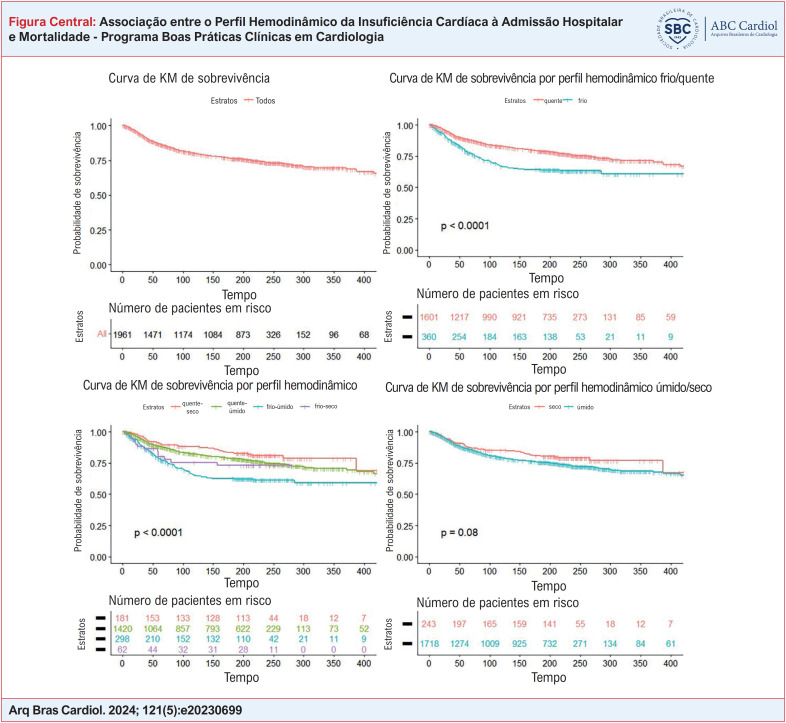
Curvas de Kaplan Meier em relação à mortalidade geral.

### Análise multivariada

Ao se comparar com o perfil de referência quente-seco, os pacientes admitidos com perfil frio-úmido apresentaram risco aumentado de óbito na internação (HR= 2,2, IC 95% 1,20-4,04), assim como perfil frio-seco (HR= 2,39; IC 95% 1,02-5,57). O perfil quente-úmido, por sua vez, não se associou ao aumento de risco de óbito na internação (HR= 1,33, IC 95% 0,76-2,33). No período de seguimento do estudo (até seis meses após a alta), esse risco aumentado de óbito também se manteve para o perfil frio-úmido (HR= 1,96, IC 95% 1,3-3,0) (Tabela 3).

**Tabela 3:** Riscos Relativos de óbito estimados pelo Modelo de Cox e razões das chances (hazard ratios) de reinternação estimadas pelo modelo de regressão logística, com intervalo de 95% de confiança, por perfil hemodinâmico (perfil de referência: quente-seco)

Perfil Hemodinâmico	Hazard ratio para óbito hospitalar- HR (IC 95%)			
	Modelo não ajustado	Modelo ajustado (sexo, idade)	Modelo ajustado (sexo, idade e comorbidades†)	Modelo ajustado (anteriores e FEVE‡)
Quente e úmido	1,35 (IC 0,78-2,35)	1,31 (0,75-2,27)	1,33 (0,76-2,31)	1,33 (0,76-2,33)
Frio e úmido*	2,21 (1,24-3,95)	2,15 (1,20-3,85)	2,18 (1,21-3,93)	2,20 (1,20 – 4,04)
Frio e seco*	2,5 (1,08- 5,77)	2,53 (1,09- 5,87)	2,37 (1,02- 5,52)	2,39 (1,02- 5,57)
**Perfil Hemodinâmico**	**Hazard ratio para óbito no seguimento de 6 meses- HR (IC 95%)**			
	**Modelo não ajustado**	**Modelo ajustado (sexo, idade)**	**Modelo ajustado (sexo, idade e comorbidades** † **)**	**Modelo ajustado (anteriores e FEVE** ‡ **)**
Quente e úmido	1,33 (0,93-1,92)	1,26 (0,87-1,82)	1,20 (0,83-1,74)	1,18 (0,82-1,72)
Frio e úmido*	2,34 (1,57- 3,5)	2,24 (1,5- 3,34)	2,02 (1,34- 3,03)	1,96 (1,3- 2,97)
Frio e seco	1,66 (0,89-3,11)	1,54 (0,82-2,89)	1,36 (0,73-2,57)	1,34 (0,71-2,53)
**Perfil Hemodinâmico**	**Odds Ratio para reinternação em 6 meses- OR (IC 95%)**			
	**Modelo não ajustado**	**Modelo ajustado (sexo, idade)**	**Modelo ajustado (sexo, idade e comorbidades** † **)**	**Modelo ajustado (anteriores e FEVE** ‡ **)**
Quente e úmido*	2,51 (1,54-4,33)	2,55 (1,56-4,42)	2,38 (1,45-4,14)	2,39 (1,41-4,05)
Frio e úmido*	2,75 (1,54-5,10)	2,81 (1,57-5,22)	2,57 (1,42-4,8)	2,59 (1,40-4,79)
Frio e seco	1,35 (0,46-3,49)	1,39 (0,47-3,61)	1,27 (0,43-3,34)	1,28 (0,47-3,51)

* p valor < 0,05 para diferença entre os perfis hemodinâmicos.

† Hipertensão arterial, doença pulmonar obstrutiva crônica, doença de Chagas, doença coronariana, diabetes, fibrilação atrial/flutter atrial);

‡ Modelos ajustados com método de imputação múltipla para os valores faltantes da variável FEVE (fração de ejeção do ventrículo esquerdo).

Além disso, comparado ao perfil de referência (quente-seco), os pacientes admitidos em perfil quente-úmido tiveram maior chance de reinternação em seis meses (OR= 2,39; IC 95% 1,41-4,05), assim como aqueles de perfil frio-úmido (OR= 2,59, IC 95% 1,40-4,79) (Tabela 3).

Avaliou-se também separadamente a perfusão (perfis frios x perfis quentes) e a congestão (perfis úmidos x perfis secos) pela análise multivariada. Para o desfecho primário de óbito na internação, os perfis frios aumentaram o risco quando comparados aos perfis quentes (HR= 1,72, IC 95% 1,27-2,31). Os perfis úmidos, por sua vez, não se associaram ao aumento de risco de óbito na internação (HR= 1,17, IC 95% 0,76-1,80). O mesmo resultado é observado na análise do período total (mortalidade hospitalar e em 6 meses de seguimento), com risco aumentado de morte para os perfis frios em relação aos perfis quentes (HR= 1,61; IC 95% 1,29-2,02) e sem aumento de risco para os perfis úmidos em relação aos secos (HR= 1,27; IC 95% 0,93-1,73).

Quanto à chance de reinternação no período em seis meses, observamos que os pacientes admitidos com perfis úmidos apresentaram risco aumentado de reinternação (OR= 2,30, IC 95% 1,45-3,65), comparado ao grupo com perfis secos. Já os perfis frios, comparados aos perfis quentes, não aumentaram o risco de reinternação com significância estatística (OR= 1,10; IC 95% 0,78-1,56).

## Discussão

Os resultados do nosso estudo trazem marcadores prognósticos para os pacientes internados por IC aguda no Brasil. As principais características desses pacientes foram renda familiar baixa, em sua maioria idosos e do sexo masculino, com múltiplas comorbidades, fração de ejeção reduzida e IC já conhecida e sintomática. A distribuição da etiologia da IC foi semelhante à descrita na literatura,^3^ mas vale destacar a alta prevalência de doença de Chagas em nosso meio, que correspondeu a quase 10% da amostra. A maior parte dos pacientes foi admitida no serviço de emergência com perfil quente e úmido. Foram encontradas altas taxas de mortalidade e reinternação hospitalar, com associação significativa ao perfil hemodinâmico de admissão.

O tempo de permanência hospitalar foi 10 dias maior que de coortes internacionais, onde o tempo médio é de aproximadamente sete dias.^3^ Esse dado é preocupante já que o estudo foi realizado em centros de referência da saúde pública brasileira e demonstra a dificuldade de otimização das terapias em tempo hábil. Isso também pode representar maior gravidade desses pacientes à admissão por dificuldade de acesso à propedêutica e tratamento adequado, considerando o contexto social da população do estudo.

Em relação à taxa de mortalidade intra-hospitalar, o estudo apresentou resultados semelhantes ao último registro brasileiro (11,1% x 12,6%).^2^ As taxas também são semelhantes aos dados existentes da mortalidade na América Latina (11,1% x 11,7%).^14^ Sabemos que os dados variam entre os centros participantes pelo perfil de pacientes admitidos em cada local. Dados recentemente publicados do Hospital das Clínicas da UFMG, em Belo Horizonte, exibem maior mortalidade hospitalar (17,9%),^15^ porém a etiologia predominante da IC foi Doença de Chagas (25,8%) e 18,3% dos pacientes foram encaminhados para transplante cardíaco na mesma internação, confirmando a maior gravidade dessa amostra.^16^

Dados da coorte do presente estudo revelam maior mortalidade hospitalar (11,1%) ao se comparar os de coortes europeias (5,5%)^3^ e americanas (4,0%).^4,17^ Ao se estratificar essa mortalidade pelo perfil hemodinâmico de admissão, encontramos taxas maiores para os perfis frios, que representam os pacientes hipotensos e mais graves, com comprometimento multiorgânico pela IC. Em nosso modelo ajustado, encontramos um aumento de risco de óbito intra-hospitalar com *hazard ratio* de 2,20 (IC 95% 1,20 – 4,04) para o perfil hemodinâmico frio-úmido. Em um registro europeu semelhante, esse perfil associou-se ao risco de óbito intra-hospitalar com *hazard-ratio* de 3,47 (IC 95 % 2,31-5,22).^5^ Diante disso, o manejo dos pacientes desse grupo deve ser imediato e eficaz, a fim de tentar reverter a evolução natural dessa apresentação, com alta chance de óbito. Times de reposta rápida em choque cardiogênico e protocolos institucionais bem elaborados podem contribuir para melhoria dos resultados.^18^

Ao se analisar a mortalidade geral do estudo, agrupando a mortalidade hospitalar e no seguimento de seis meses, também há diferença estatística entre os perfis, com maior taxa de mortalidade para o perfil frio-úmido (32%). Esse perfil apresentou maior risco de morte no seguimento, com *hazard-ratio* de 1,96 (IC 95% 1,3-2,97). Dados europeus corroboram esses achados, com taxas de mortalidade geral em um ano de 26,7% após internação hospitalar por IC aguda, sendo de 54% nos pacientes admitidos em choque cardiogênico.^3^

Outro achado do estudo foi a maior chance de reinternação dos pacientes com perfis úmidos (OR = 2,30; IC 95% 1,45-3,65), que apresentaram sintomas congestivos recorrentes no seguimento. É descrito na literatura que os perfis mais congestos têm maior taxa de reinternação em um ano, sendo de 32,2% para perfil frio-úmido, 26,9% para perfil quente-úmido e somente 16,4% para perfil quente-seco (p<0,001)^3^. Diferentemente dos perfis quentes, os perfis frios isoladamente não se associaram ao aumento de reinternações (OR= 1,10, IC 95% 0,78-1,56), o que pode estar associado à maior mortalidade intra-hospitalar nesses perfis.

Há um período de vulnerabilidade nos 30 dias após a alta, em que o acompanhamento é importante para reavaliar sintomas congestivos ou outras complicações, a fim de prevenir reinternações. Estratégias de otimização e monitorização do tratamento devem ser traçadas antes da alta e para o seguimento ambulatorial. Essas medidas envolvem desde avaliação multidisciplinar: orientações dietéticas, aderência medicamentosa e programas de reabilitação cardíaca, até avaliação médica periódica quanto ao ajuste e tolerância a medicações, monitorização de biomarcadores e sintomas congestivos.^19^ Como exemplo, o estudo multicêntrico randomizado STRONG-HF^20^ comparou uma estratégia mais intensiva de seguimento ambulatorial após internação por IC aguda, com titulação das medicações modificadoras de doença (betabloqueadores, bloqueadores do sistema renina-angiotensina e antagonistas dos receptores mineralocorticoides) até doses-alvo em duas semanas após a alta, em vez do manejo habitual, e demonstrou redução de reinternação ou óbito em 6 meses de 23% para 15% (p = 0,002).

O presente estudo apresenta limitações por se tratar de uma coorte, estudo de não intervenção, em que as associações entre perfil hemodinâmico e desfechos podem conter vieses. As perdas por falhas de registro ou seguimento foram altas, mas todas consideradas nas análises estatísticas. A informação sobre a reinternação em seis meses foi coletada por ligação telefônica, de forma categórica (presente ou ausente) e isso limitou a melhor caracterização desse desfecho. Além disso, a classificação do perfil hemodinâmico foi realizada pelo julgamento clínico do médico emergencista, o que traduz subjetividade, mas se aproxima mais da realidade. O estudo foi realizado em hospitais terciários, a maioria também universitários, que são centros de referência regionais. Se as Unidades de Pronto Atendimento (serviço médico de urgência pré-hospitalar no sistema de saúde brasileiro) e hospitais de baixa complexidade fossem avaliados no estudo, nossos resultados poderiam ser diferentes.

## Conclusão

A IC aguda no Brasil apresenta alta morbimortalidade, que se relaciona com o perfil hemodinâmico de admissão hospitalar. Os pacientes admitidos em perfil frio (mal perfundidos) tiveram um risco de óbito aumentado em 72% na internação e em 61% no seguimento de seis meses. Pacientes que se apresentavam congestos (perfil úmido) têm 2,3 mais chances de reinternação em seis meses.

## *Material suplementar

Para informação adicional, por favor, clique aqui.
