# Supplementary material for: Associação entre o Perfil Hemodinâmico da Insuficiência Cardíaca à Admissão Hospitalar e Mortalidade - Programa Boas Práticas Clínicas em Cardiologia
Source: Arq Bras Cardiol. 2024 Jun 6;121(5):e20230699. [Article in Portuguese] doi: 10.36660/abc.20230699 (PMC11216337; doi:10.36660/abc.20230699)
Supplement: Translation

# Translation: Association between Hemodynamic Profile at Hospital Admission and Mortality in Acute Heart Failure Patients Included in the Best Practice in Cardiology Program in Brazil

**DOI:** 10.36660/abc.20230699i

**Authors:** André Silva Rodrigues, Fábio Morato de Castilho, Aloisio Joaquim Freitas Ribeiro, Luiz Guilherme Passaglia, Fabio Papa Taniguchi, Antonio L. Ribeiro

**Affiliations:** 1 Belo Horizonte MG Brazil Universidade Federal de Minas Gerais - Serviço de Cardiologia HC- UFMG, Belo Horizonte, MG – Brazil; 2 Belo Horizonte MG Brazil Universidade Federal de Minas Gerais - Departamento de Clínica Médica - Faculdade de Medicina – UFMG, Belo Horizonte, MG – Brazil; 3 Belo Horizonte MG Brazil Universidade Federal de Minas Gerais - Departamento de Estatística, Belo Horizonte, MG – Brazil; 4 São Paulo SP Brazil Hospital do Coração, São Paulo, SP – Brazil; 5 Belo Horizonte MG Brazil Universidade Federal de Minas Gerais - Centro de Telessaúde - Hospital das Clínicas – UFMG, Belo Horizonte, MG – Brazil

**Keywords:** Heart Failure, Mortality, Hospitalization

## Abstract

**Background:**

Heart failure (HF) contributes to a high burden of hospitalization, and its form of presentation is associated with disease prognosis.

**Objectives:**

To describe the association of hemodynamic profile of acute HF patients at hospital admission, based on congestion (wet/dry) and perfusion (cold/warm), with mortality, hospital length of stay and risk of readmission.

**Methods:**

Cohort study, with patients participating in the "Best Practice in Cardiology" program, admitted for acute HF in Brazilian public hospitals between March 2016 and December 2019, with a six-month follow-up. Characteristics of the population and hemodynamic profile at admission were analyzed, in addition to survival analysis using Cox proportional hazard model for associations between hemodynamic profile at admission and mortality, and logistic regression for the risk of rehospitalization, using a statistical significance level of 5%.

**Results:**

A total of 1,978 patients were assessed, with mean age of 60.2 (±14.8) years and mean left ventricular ejection fraction of 39.8% (±17.3%). A high six-month mortality rate (22%) was observed, with an association of cold hemodynamic profiles with in-hospital mortality (HR=1.72, 95%CI 1.27-2.31; p < 0.001) and six-month mortality (HR= 1.61, 95%CI 1.29-2.02). Six-month rehospitalization rate was 22%, and higher among patients with wet profiles (OR 2.30; 95%CI 1.45-3.65; p < 0.001).

**Conclusions:**

Acute HF is associated with high mortality and rehospitalization rates. Patient hemodynamic profile at admission is a good prognostic marker of this condition.

## Introduction

Heart failure (HF) is a complex syndrome that causes high admission and mortality rates. Among cardiovascular diseases, HF is the main cause of hospitalization in Brazil, accounting for more than three million admissions over the last 10 years.^1^ In-hospital mortality among patients admitted for acute HF is still high – 12.6% in 2015 BREATHE registry.^2^

International registries, however, present lower mortality rates, 5.5% during hospitalization and 26.7% one-year post-discharge in Europe.^3^ In the large ADHERE registry,^4^ mean hospital mortality rate for acute HF was 3.5%. These rates vary according to clinical profiles based on congestion and perfusion at admission, with an increase of up to 66% in the likelihood of death in patients with a cold-wet profile as compared with those with a warm-dry profile.^5^

Clinical assessment of perfusion (blood pressure, pulse pressure, peripheral perfusion, altered consciousness) and congestion (increased jugular venous pressure, orthopnea, edema, B3) determines patient hemodynamic profile: warm-dry, warm-wet, cold-dry and cold-wet, which is associated with disease severity and prognosis.^6^ This classification was first described by Diamond-Forrester^7^ in 1976, for patients who had suffered acute myocardial infarction. In the context of HF, Stevenson LW^8,9^ published several studies on the importance of recognizing clinical signs of congestion and hypoperfusion in patients admitted with acute HF to receive therapy guided by the hemodynamic profile, prioritizing diuretic therapy and vasodilation in patients with wet profile and inotropic therapy for patients with cold profiles.^8,9^

It is expected that the hemodynamic profile at admission will be a good prognostic marker of acute HF in Brazil and help to characterize the current behavior of the disease in the country. By associating the hemodynamic profile at admission with the outcomes mortality and risk of rehospitalization, specific interventions to each profile may be used to achieve better results.

## Objectives

The aim of the present study was to demonstrate the association between patient hemodynamic profile at hospital admission and major cardiovascular outcomes – overall in-hospital mortality (primary outcome) and six-month mortality (in-hospital and during follow-up); hospital length of stay and six-month readmission rate (secondary outcomes).

## Methods

This was cohort study, with patients admitted for acute HF in 17 public hospitals in Brazil from March 2016 to December 2019. These institutions are tertiary hospitals located in the five Brazilian geographic regions, participants of the Brazilian Society of Cardiology guideline for Best Practice in Cardiology (BPC). The BPC is inspired in the American Heart Association's Get With The Guidelines (GWTG) and aims to assess adherence rates to performance indicators recommended by the Brazilian and the American guidelines. The BPC design and results were described in previous publications.^10-12^

### Population

Patients aged 18 years or older, admitted with acute HF (IDC 10 I50; I50.0; I50.1 or I50.9) in the participating hospitals were included. Patients who would be transferred to other institutions, those with an expected length of stay less than 24 hours, and HF patients with other possible causes of dyspnea were excluded.

### Data collection

Data were collected prospectively during hospitalization, using electronic medical records and specific forms, and by telephone interview administered by trained interviewers at 30 days and six months after discharge. All participants signed an informed consent form, and the study was approved by the ethics committee of the coordinating center – HCor Sao Paulo – (approval number 48561715.5.1001.0060) and the ethics committee of UFMG General Hospital (approval number 1.487.029).

### Hemodynamic profiles

Patient hemodynamic profile was clinically determined by the doctor in charge of patient admission. The profiles were defined as: dry-warm (no signs of decompensation); warm-wet (well perfused but congested); warm-wet (well perfused, but congested); cold-dry (poor peripheral perfusion, but no congestion); cold-wet (poor perfusion and congested). In this classification into four categories, there are two levels of exposure: perfusion (cold or warm) and congestion (wet or dry). Based on this, survival analysis and multivariate analysis were performed to estimate the risks for the outcomes of interest for the four classical profiles, as well as for the perfusion (cold profiles x warm profiles) and congestion (wet profiles x dry profiles) patterns.

### Statistical analysis

In the descriptive analysis of the outcome variables and the covariable of interest by hemodynamic profiles, continuous variables were described as mean ± standard deviation or median and interquartile range (Q1 and Q3) according to data normality (Shapiro Wilk test). Categorical variables were described as absolute and relative frequencies. The hemodynamic profiles were compared by the chi-square test (categorical variables) and by the Kruskal-Wallis test (quantitative variables). Multiple comparisons were made by the Dunn test (quantitative variables) and the Z test for two proportions (categorical variables), using the Holm correction method. These analyses were made using the rstatix package of the R software.

Survival curves were constructed using the Kaplan-Meier method, by univariate analysis, to evaluate the effect of the hemodynamic profile (in four and two categories) at admission on mortality during the study follow-up period – six months (log-rank test). The reference group for calculation of risks and chances was the warm-dry group.

The Cox proportional hazards model was used to investigate the influence of the hemodynamic profiles on mortality during hospitalization and on six-month follow-up, controlling the effects of age, sex, presence of comorbidities at admission and left ventricular ejection fraction (LVEF), in an incremental way. To assess the influence of the hemodynamic profiles on the occurrence of at least one readmission in six months, regression logistic models were used, with the same control variables included in the Cox model, in addition to the follow-up period after discharge.

The significance level was set at 5%. All analyses were performed using the stats, rstatix, survival, survminer, and mice packages of the R software (R Core Team, 2020).

### Treatment of missing data

The hemodynamic profile of 784 (28%) patients was not informed, who were then excluded from the study. Comparison of the groups with and without information on the hemodynamic profile regarding the primary outcome (death during hospitalization) did not reveal a significant difference between them (p=0.08; Supplementary Table 1), corroborating the assumption of random data loss.

Among the patients with valid information about the hemodynamic profile, LVEF values of 96 were missing. These missing variables were imputed using the multiple imputation method (pmm, predictive mean matching), available in the MICE (Multivariate Imputation by Chained Equation) package of the R software,^13^ resulting in 10 complete sets. In this imputation model, the variables sex, age, presence of comorbidities, hemodynamic profile and estimate of the risk of death during hospitalization (Nelson Aalen method) were used. In addition, data on medication of 679 patients were missing. Since this information was used in the sample description only, these values were not imputed.

For the secondary outcome (readmission within six months), 1,543 patients were assessed, consisting of 215 participants where two telephone contacts were unsuccessful during this period subtracted from 1,758 survivors (12% of loss to follow-up).

## Results

### Description of the sample at admission

Of the 2,762 patients included in the cohort until 2019, the analysis included data from 1,978 patients with data on hemodynamic profile available on their medical records. Baseline data are described in Table 1, stratified by the hemodynamic profile at admission. Mean age of the study population was 60 years; most patients were male, with low educational attainment (65% illiterate or elementary school) and low income (73.2% receiving up to two minimum wages of family income). The most common comorbidities reported by the patients at admission were hypertension, diabetes mellitus and atrial fibrillation.

**Table 1:** Baseline characteristics of patients, stratified by hemodynamic profile

Baseline characteristics	Total (N=1978)	Warm-dry (n=183)	Warm-wet (n=1435)	Cold-wet (n=298)	Cold-dry (n=62)
Gênero masculino* - n (%)	1154 (58.3)	91 (49.7)	832 (58)	191 (64.1)	40 (64.5)
Mean age (SD)	60.2 (14.8)	54.8 (16.7)	61.1 (14.4)	59.2 (14.9)	61.8 (14.4)
**Educational attainment** * **- n (%)**					
Illiterate	192 (9.7)	16 (8.7)	152 (10.6)	21 (7)	3 (4.8)
Elementary school	1094 (55.3)	85 (46.4)	817 (56.9)	157 (52.7)	35 (56.5)
High school	519 (26.2)	65 (35.5)	353 (24.6)	81 (27.2)	20 (32.3)
Higher education	169 (8.5)	17 (9.3)	112 (7.8)	36 (12.1)	4 (6.5)
**Family income** * **- n (%)**					
<1 minimum wage (U$ 270)	656 (33.2)	82 (44.8)	467 (32.5)	93 (31.2)	14 (22.6)
1 - 2 wages (U$ 270- 540)	792 (40)	53 (29)	608 (42.4)	108 (36.2)	23 (37.1)
2 - 5 wages (U$ 540- 1350)	433 (21.9)	39 (21.3)	301 (21)	71 (23.8)	22 (35.5)
> 5 wages (> U$ 540)	90 (4.6)	8 (4.4)	57 (4)	22 (7.4)	3 (4.8)
**Self-reported comorbidities, n (%)**					
Arterial hypertension*	1351(68.3)	111 (60.7)	1028 (71.6)	178 (59.7)	34 (54.8)
Diabetes mellitus	652 (33)	48 (26.2)	497 (34.6)	92 (30.9)	15 (24.2)
Chagas disease*	208 (10.5)	12 (6.6)	135 (9.4)	51 (17.1)	10 (16.1)
Coronary artery disease	285 (14.4)	20 (10.9)	217 (15.1)	42 (14.1)	6 (9.7)
Atrial fibrillation/ flutter*	503 (25.4)	30 (16.4)	375 (26.1)	75 (25.2)	23 (37.1)
Chronic kidney disease*	309 (15.6)	8 (4.4)	227 (15.8)	60 (20.1)	14 (22.6)
**Functional class** * **n (%)**					
I-II	208 (10.5)	67 (36.6)	114 (7.9)	18 (6)	9 (14.5)
III-IV	1393 (70.4)	102 (55.7)	1032 (71.9)	212 (71.1)	47 (75.8)
Not informed	377 (19.1)	14 (7.7)	289 (20.1)	68 (22.8)	6 (9.7)
**Ejection fraction** * **n (%)**					
> 50%	509 (25.7)	77 (42.1)	383 (26.7)	37 (12.4)	12 (19.4)
41-50%	232 (11.7)	24 (13.1)	173 (12.1)	23 (7.7)	12 (19.4)
≤ 40%	1135 (57.4)	66 (36.1)	808 (56.3)	224 (59.7)	37 (59.7)
Previous heart failure – n (%)	1578 (79.8)	147 (80.3)	1131 (78.9)	243 (81.5)	57 (79.8)
Hospitalizations in the last six months*- n (%)	651 (32.9)	65 (35.5)	444 (30.9)	124 (41.7)	18 (29.1)
**Previous Medications - n (%)**†					
Betablockers*	926 (71.3)	75 (61)	676 (71)	147 (77)	28 (84.8)
ACEI or ARB	854 (65.7)	80 (65)	616 (64.7)	137 (71.7)	21 (63.6)
Spironolactone*	524 (40.3)	45 (36.6)	348 (36.6)	109 (57.1)	22 (66.7)
Loop diuretics*	885 (68.1)	72 (58.5)	648 (68.1)	137 (71.7)	28 (84.8)

* p value < 0.05 for differences between the hemodynamic profiles (chi-square test);

† N= 1,299 for missing data about the use of previous medications; ACEI: angiotensin converting enzyme inhibitors; ARB: angiotensin receptor blocker.

Most patients already had a previous HF diagnosis (decompensated HF) and had a New York Association (NYHA) functional class III-IV. The most frequent etiologies of HF during hospitalization (defined after tests, by an assistant staff) were idiopathic (23.3%), ischemic (21.3%), hypertensive (16.1%), valvar (15.3%) and Chagas cardiomyopathy (9.9%), among others (14.1%) (Supplementary Table 2). Most patients had HF with reduced LVEF (≤ 40%), with a mean of 39.8% (±17,3%), and the most frequent hemodynamic profile at admission was the warm-wet profile.

### Analysis of the outcomes of interest

A total of 220 in-hospital deaths occurred, corresponding to an overall mortality of 11.1%. In-hospital mortality rate was higher in patients admitted for poor peripheral perfusion (cold profile) – 21.5% for the cold-wet profile and 14.5% for the cold-dry profile. Only 10 patients (0.5% of the sample) underwent heart transplantation during hospitalization. During the study period (hospitalization and six-month follow-up), overall mortality rate was 22%, 32% in patients with a cold-wet profile. In addition, hospital length of stay varied significantly across the hemodynamic profiles, with a median of 17 (9-33) in the general population and 23 (13-42) days in the cold-wet profile (Table 2).

**Table 2:** Primary and secondary outcomes by hemodynamic profile

Outcomes	Total (N=1978)	Warm-dry Group 1 (n=183)	Warm-wet Group 2 (n=1435)	Cold-wet Group 3 (n=298)	Cold-dry Group 4 (n=62)	p-value
In-hospital deaths N (%)	220 (11.1)	14 (7.7)	133 (9.3)	64 (21.5)	9 (14.5)	<0.001 (1=2≠3=4)
Overall mortality (hospitalization + 6- month follow-up)- N (%)	432 (22)	32 (16)	290 (20)	96 (32)	14 (22)	<0.001 (1=2≠3=4)
Days of hospitalization Median (interquartile range)	17 (9-33)	22 (10-35)	16 (8-30)	23 (13-42)	15 (10-32)	<0.001 (1≠2=3=4)
Rehospitalization*- N(%)	334 (22)	18 (11.5)	262 (23)	48 (23.8)	6 (13)	0.004 (1≠2=3≠4)

* N=1543 due to loss to follow-up (deaths during hospitalization and follow-up losses).

In the six-month period after hospital discharge, 1,543 patients were contacted twice by telephone. Of these patients, 334 (22%) patients were readmitted at least once, and a significant effect of the hemodynamic profile at admission was observed on this outcome. In this regard, rehospitalization was more frequent in congested patients (wet profiles), 23% in the warm-wet group, and 23.8% in the cold-wet group, and only in 11.5% and 10.9% in the warm-dry and cold-dry profiles, respectively (Table 2).

In the univariate survival data analysis using Kaplan-Meier curves, an association between clinical profile at admission and mortality in the whole study period (hospitalization + six-month follow-up) was observed, with a higher mortality among patients with the cold-wet profile as compared with the others (p < 0.0001, log-rank test). Analysis of congestion and perfusion alone revealed that cold profiles were associated with higher mortality in the follow-up than warm profiles (p < 0.0001, log-rank test), which was not observed in wet *versus* dry profiles (p = 0.08, log-rank test) (Central Illustration).

**Figure:** Kaplan-Meier (KM) curves for overall mortality.

### Multivariate analysis

Patients admitted with a cold-wet profile and patients with a cold-dry profile showed an increased risk of in-hospital death as compared with those with a warm-dry profile (HR= 2.2, 95%CI 1.20-4.04 and 2.39, 95%CI 1.02-5.57, respectively). On the other hand, the warm-wet profile was not associated with an increased risk of death (HR= 1.33; 95%CI 0.76-2.33). In the study follow-up period (up to six months after discharge), this increased risk of death was also observed in the cold-wet profile (HR= 1.96, 95%CI 1.3-3.0) (Table 3).

**Table 3:** Relative risks of death estimated by Cox model and hazard ratios for readmissions estimated by logistic regression model, with 95% confidence interval, by hemodynamic profile (reference profile: warm-dry)

Hemodynamic profile	Hazard ratio for in-hospital mortality (95%CI)			
	Unadjusted model	Adjusted model (sex, age)	Adjusted model (sex, age, comorbidities†)	Adjusted model (previous and LVEF‡)
Warm and wet	1.35 (IC 0.78-2.35)	1.31 (0.75-2.27)	1.33 (0.76-2.31)	1.33 (0.76-2.33)
Cold and wet*	2.21 (1.24-3.95)	2.15 (1.20-3.85)	2.18 (1.21-3.93)	2.20 (1.20 – 4.04)
Cold and dry*	2.5 (1.08- 5.77)	2.53 (1.09- 5.87)	2.37 (1.02- 5.52)	2.39 (1.02- 5.57)
**Hemodynamic profile**	**Hazard ratio of death during the six-month follow-up period - HR (95%CI)**			
	**Unadjusted model**	**Adjusted model (sex, age)**	**Adjusted model (sex, age, comorbidities** † **)**	**Adjustedmodel (previous and LVEF** ‡ **)**
Warm and wet	1.33 (0.93-1.92)	1.26 (0.87-1.82)	1.20 (0.83-1.74)	1.18 (0.82-1.72)
Cold and wet*	2.34 (1.57- 3.5)	2.24 (1.5- 3.34)	2.02 (1.34- 3.03)	1.96 (1.3- 2.97)
Cold and dry	1.66 (0.89-3.11)	1.54 (0.82-2.89)	1.36 (0.73-2.57)	1.34 (0.71-2.53)
**Hemodynamic profile**	**Odds ratio of readmission in six months - OR (95%CI)**			
	**Unadjusted model**	**Adjusted model (sex, age)**	**Adjusted model (sex, age, comorbidities** † **)**	**Adjusted model (previous and LVEF** ‡ **)**
Warm and wet*	2.51 (1.54-4.33)	2.55 (1.56-4.42)	2.38 (1.45-4.14)	2.39 (1.41-4.05)
Cold and wet *	2.75 (1.54-5.10)	2.81 (1.57-5.22)	2.57 (1.42-4.8)	2.59 (1.40-4.79)
Cold and dry	1.35 (0.46-3.49)	1.39 (0.47-3.61)	1.27 (0.43-3.34)	1.28 (0.47-3.51)

* p value < 0.05 for differences between the hemodynamic profiles;

† Hypertension, chronic pulmonary obstructive disease, Chagas disease, coronary artery disease, diabetes, atrial fibrillation/atrial flutter);

‡ Models adjusted by multiple imputation of missing LVEF (left ventricular ejection fraction) values.

In addition, when compared with the warm-dry profile, patients admitted with a warm-wet profile (OR= 2.39; 95%CI 1.41-4.05) and those with a cold-wet profile (OR= 2.59; 95%CI 1.40-4.79) had a higher risk of readmission in six months (Table 3).

We also evaluated perfusion (cold vs. warm) and congestion (wet vs. dry) separately by multivariate analysis. For the primary outcome, cold profiles increased the risk of in-hospital death when compared to warm profiles (HR= 1.61; 95%CI 1.29-2.02), whereas wet profiles did not increase this risk as compared with dry profiles (HR= 1.27; 95%CI 0.93-1.73).

Regarding the risk of readmission in six months, we found that patients admitted with a wet profile showed an increased risk of readmission (OR= 2.30, 95%CI 1.45-3.65), as compared with those admitted with dry profiles. On the other hand, warm profiles were not significantly associated with an increased risk of rehospitalization (OR= 1.10; 95%CI 0.78-1.56).

## Discussion

Results of the present study reveal prognostic markers for patients admitted for acute HF in Brazil. Main characteristics of these patients were low family income, mostly older males with multiple comorbidities, reduced ejection fraction and known and symptomatic HF. Distribution of HF etiology was similar to that described in the literature;^3^ it is worth mentioning, however, the high prevalence of Chagas disease in our country, which accounted for almost 10% of the sample. Most patients were admitted to the emergency department with a warm and wet profile. High mortality and rehospitalization rates were found, with a significant association with hemodynamic profile at admission.

Hospital length of stay was 10 days longer than that reported in international cohorts (mean of seven days).^3^ This information is alarming, since the study was conducted in renowned public health centers in Brazil and reflect the difficulty in providing optimal treatment in a timely manner. This could also represent higher severity of these patients at admission due to limited access to diagnostic tests and appropriate treatment, considering the social context of the study population.

In-hospital mortality rate of the present study was similar to that reported in the last Brazilian registry (11.1% x 12.6%).^2^ This was also similar to mortality data available from the Latin America (11.1% x 11.7%).^14^ We do know that data may vary between participating centers according to the profile of patients at admission. Recently published data from the UFMG general hospital, in Belo Horizonte, Brazil, showed a higher mortality rate (17.9%),^15^ though the main cause of HF was Chagas disease (25.8%) and 18.3% of patients were referred for heart transplantation in the same hospitalization, confirming the severity of this sample.^16^

The cohort of the present study showed a higher in-hospital mortality (11.1%) when compared with European (5.5%)^3^ and American (4.0%)^4,17^ cohorts. When mortality was stratified by patients’ hemodynamic profile at admission, cold profile has higher mortality, which represents hypotensive and more severe patients, with multiple organ failure due to HF. In our adjusted model, we found an increased risk of in-hospital death, with a hazard ratio of 2.20 (95%CI 1.20 – 4.04) for the cold-wet profile. In a similar European registry, this profile was associated with in-hospital death, with a hazard ratio of 3.47 (95%CI 2.31-5.22).^5^ Therefore, management of these patients should be immediate and effective, in order to reverse the natural course of the cold-wet profile, with a high risk of death. Rapid response teams for cardiogenic shock and well-designed institutional protocols can contribute to better outcomes.^18^

In the analysis of overall mortality, in-hospital and six-month mortalities were grouped, revealing a statistically significant difference between the profiles, with a higher mortality for the cold-wet profile (32%). In addition, this profile showed a higher follow-up mortality risk, with a hazard ratio of 1.96 (95%CI 1.3- 2.97). European data corroborate these findings, showing a one-year mortality rate of 26.7% after hospital admission for acute HF, and 54% among patients admitted with cardiogenic shock.^3^

Another finding of this study was the higher chance of readmission in patients with wet profiles (OR = 2.30; 95%CI 1.45-3.65), who developed recurrent congestive symptoms in the follow-up. Patients with a "more congested" profile have been associated with higher one-year rehospitalization rates – 32.2% for the cold-wet profile, 26.9% for the warm-wet and 16.4% for the warm-dry profile (p<0.001).^3^ Unlike warm profiles, cold profiles were not associated with higher rehospitalization (OR= 1.10, 95%CI 0.78-1.56), which may be associated with the higher in-hospital mortality in these profiles.

The 30-day period following discharge is characterized by vulnerability, during which the follow-up is crucial to reassess congestive symptoms and other complications to prevent rehospitalizations. Optimization strategies and treatment monitoring should be planned before discharge, targeting outpatient follow-up. These measures include a multidisciplinary approach: dietary counseling, adherence to drug therapy, cardiac rehabilitation programs, periodical medical examination for adjustment and tolerance to drug treatment, monitoring of biomarkers and congestive symptoms.^19^ For example, the randomized multicentric study STRONG-HF^20^ compared a more intensive strategy of outpatient follow-up after hospitalization for acute HF, consisting of up-titration of disease-modifying agents (betablockers, renin- angiotensin system blockers and mineralocorticoid receptor antagonists) to target doses within two weeks of discharge instead of usual care, and showed a reduction in readmission and six-month mortality from 23% to 15% (p=0.002).

Limitations of the present study include the fact that it was a cohort, non-interventional study, and hence associations between hemodynamic profile and outcomes may be biased. Although losses due to reporting failure and losses to follow-up were high, all were considered in the statistical analysis. Information on readmission within six months was collected by telephone, in a categorical manner – present or absent – which precluded a better characterization of this outcome. In addition, classification of the hemodynamic profile was made at the emergency physician's discretion, which, despite subjective, is closer to reality. Also, the study was carried out in tertiary care hospitals that are local referral centers. Probably, if emergency and primary care centers, as well as secondary care hospitals were included, our results may have been different.

## Conclusion

Acute HF has high morbidity and mortality in Brazil and is related to the hemodynamic profile of patients at admission. Patients admitted with a cold profile (poorly perfused) had a 72% increased risk of in-hospital death and a 61% increased risk of death during the six-month follow-up. Congested patients (we profile) had 2.3 times greater chance of readmission in six months.

## *Supplemental Materials

For additional information, please click here.
